# Publisher’s Note – Received Date Error, *Int. J. Environ. Res. Public Health* 2009, *6*, 3105–3114

**DOI:** 10.3390/ijerph7010028

**Published:** 2009-12-29

**Authors:** Shu-Kun Lin

**Affiliations:** Molecular Diversity Preservation International (MDPI), Kandererstrasse 25, CH-4057 Basel, Switzerland; E-Mail: lin@mdpi.org

*Publisher’s Note* added on 29 December 2009: The received date of *Int. J. Environ. Res. Public Health* **2009**, *6*, 3105–3114 was wrongly published. The correct received date is 13 October 2009, not 13 October 2008.
